# Supplementary material for: Assessing potential added benefits of trauma-focused content to a guided low-intensity psychoeducational intervention for perinatal women: A propensity score-matched analysis of a nonrandomized trial
Source: Glob Ment Health (Camb). 2025 Dec 19;13:e14. doi: 10.1017/gmh.2025.10094 (PMC12835942; doi:10.1017/gmh.2025.10094)
Supplement: Miller-Graff et al. supplementary material [file S2054425125100940sup001.docx]

*Electronic Supplement 1.*

Baseline differences between conditions in the unmatched sample

|  | Perinatal Health | | Trauma-focused Perinatal Health | |  |
| --- | --- | --- | --- | --- | --- |
|  | M | SD | M | SD | Significant difference (t, *p*) |
| ACEs | 2.74 | 2.02 | 3.56 | 2.30 | -2.31,  *p* = .016 |
| IPV | 0.76 | 0.11 | 0.12 | 0.15 | -1.98  *p =* .049 |
| Depression | 13.46 | 8.82 | 16.63 | 1.20 | *-*2.04  *p* = .043 |
| Posttraumatic stress | 11.57 | 10.15 | 14.92 | 12.53 | 1.83  *p* = .069 |
| Multisystem resilience | 107.74 | 14.71 | 105.61 | 17.10 | 0.84  *p* = .401 |
| Parenting confidence | 38.58 | 4.02 | 38.44 | 3.49 | 0.22  *p* = .223 |

*Note.* At the start of the pandemic, due to ongoing modifications to study design, only the perinatal health condition was implemented. Baseline differences between conditions represent those present once both were in the active implementation phase.
